# Impact of extracellular vesicles on the pathogenesis, diagnosis, and potential therapy in cardiopulmonary disease

**DOI:** 10.3389/fphar.2023.1081015

**Published:** 2023-02-20

**Authors:** Yixel M. Soto-Vázquez, Kristopher R. Genschmer

**Affiliations:** Department of Medicine, Division of Pulmonary, Allergy & Critical Care Medicine, University of Alabama at Birmingham, Birmingham, AL, United States

**Keywords:** extracellular vesicles, therapeutic, cardiovascular diseases, pulmonary diseases, COPD, CF, MSc

## Abstract

Cardiopulmonary diseases span a wide breadth of conditions affecting both heart and lung, the burden of which is globally significant. Chronic pulmonary disease and cardiovascular disease are two of the leading causes of morbidity and mortality worldwide. This makes it critical to understand disease pathogenesis, thereby providing new diagnostic and therapeutic avenues to improve clinical outcomes. Extracellular vesicles provide insight into all three of these features of the disease. Extracellular vesicles are membrane-bound vesicles released by a multitude, if not all, cell types and are involved in multiple physiological and pathological processes that play an important role in intercellular communication. They can be isolated from bodily fluids, such as blood, urine, and saliva, and their contents include a variety of proteins, proteases, and microRNA. These vesicles have shown to act as effective transmitters of biological signals within the heart and lung and have roles in the pathogenesis and diagnosis of multiple cardiopulmonary diseases as well as demonstrate potential as therapeutic agents to treat said conditions. In this review article, we will discuss the role these extracellular vesicles play in the diagnosis, pathogenesis, and therapeutic possibilities of cardiovascular, pulmonary, and infection-related cardiopulmonary diseases.

## 1 Introduction

Extracellular vesicles (EVs) are membrane-bound vesicles released by cells into the extracellular space (Schorey et al., 2015; Yáñez-Mó et al., 2015). Their membranes typically consist of a lipid bilayer, similar to the cell membrane of their origin. The content that EVs carry includes lipids, nucleic acids, and protein from the donor cell (Zaborowski et al., 2015). They are found in most, if not all, bodily fluids including blood, urine, saliva, breast milk, cerebrospinal fluid, and semen (Colombo et al., 2014). Based on their composition and biogenesis EVs are usually divided into three main subtypes: microvesicles, exosomes, and apoptotic bodies (Schorey et al., 2015). Microvesicles are formed by direct outward budding of the plasma membrane, their size goes around 100 nm (nm) to 1 μm (μm) in diameter (Zaborowski et al., 2015; Doyle and Wang, 2019). Exosomes or intraluminal vesicles (ILVs) are released by multivesicular bodies as a result of the fusion with the plasma membrane (van Niel et al., 2018), their individual diameters ranging from 30 to 150 nm (Doyle and Wang, 2019; de Abreu et al., 2020). Apoptotic bodies are released into the extracellular space when cells are dying, creating EV particles whose size ranges from 50 nm to 5000 nm in diameter (Doyle and Wang, 2019). Since assigning EVs to a particular biogenesis pathway can be difficult, the International Society of Extracellular Vesicles (ISEV) encourages authors to define EVs considering their a) physical characteristics such as size (small, medium/large) or density (low, middle, high); b) biochemical composition (e.g., CD63+/CD81+-EVs, annexin, etc.); or c) conditions of cell origin (podocyte, hypoxic, oncosomes, apoptotic bodies) rather than using the term exosome or microvesicles (Théry et al., 2018). For this review we are going refer to all extracellular vesicle as EVs unless specified otherwise.

Extracellular vesicles are involved in physiological processes such as immune regulation (Robbins and Morelli, 2014), wound healing (Ferreira et al., 2017) and supporting heart repair (Ibrahim et al., 2014). They are involved in pathological disorders such as cardiovascular disease (Ailawadi et al., 2015) and inflammation (Kulshreshtha et al., 2013). EVs derived from tumor cells have been observed to generate a microenvironment that allows tumor growth and metastasis (Kosaka et al., 2016). They also have a role in the pathogenesis of autoimmune diseases including multiple sclerosis, Type 1 diabetes and rheumatoid arthritis(Wu W. C. et al., 2020).

On the other side, EVs are considered a mechanism of intercellular communication (Raposo and Stoorvogel, 2013) maintaining tissue homeostasis by exchanging proteins, lipids (van Niel et al., 2018), mRNA and microRNA (miRNA) (Valadi et al., 2007). miRNAs are small endogenous, non-coding RNAs, which consist of 20–22 nucleotides in length (Bartel, 2004). They can be functionally delivered to cells and their role is consider as a modulator of gene expression in immune cells (Long et al., 2018). In EVs, miRNA showed immune functions as well. EVs have also emerged as a source of a biomarker in various diseases and may serve as therapeutic approaches in anti-tumor, regenerative therapies, and drug delivery (Lener et al., 2015). EVs containing a variety of different miRNAs, have been associated with multiple cardiovascular and pulmonary diseases, and function as vectors affecting gene-regulatory function (Diehl et al., 2012; Vituret et al., 2016). miRNAs provide posttranscriptional regulation of gene expression and play a crucial role in cardiovascular health and disease (Small et al., 2010). Therefore, EVs hold a potential to diagnose, treat and understand the pathogenesis of cardiovascular and lung diseases. This review, discuss the roles of EVs in diagnostic and therapeutic implications (Table 1) in cardiac and pulmonary diseases.

**TABLE 1:** Extracellular vesicles-based therapeutic in cardiopulmonary disease.

Disease	Origin of EVs	Functional contents	Therapeutic function	Reference
Atherosclerosis	Bone marrow derived macrophages	miRNA-99a, miRNA-146b, miRNA-378a	Anti-inflammation	Bouchareychas et al. (2020)
	ADSCs, MSC	Smad2/3 siRNA	Restoration of elasticity of the wall of the thoracic aorta. Decrease in expression of structural and inflammatory markers COL1A1, α-SMA, Cx43, VCAM-1, and MMP-2	Comariţa et al. (2022)
	M2 engineered macrophages	HAL	Anti-inflammation effects and alleviate atherosclerosis	Wu et al. (2020a)
Myocardial infarction	Macrophage	miR-150	Regulate TP53-IGF-1 signalling pathway, cardioprotective effect	Zheng et al. (2020)
	Embryonic stem cells	miR-294	Cell cycle progression, cell proliferation, cell survival	Khan et al. (2015)
Heart failure	Healthy donor cardiac stromal cells	miR-21-5p	Regulate apoptosis, enhance angiogenesis, cardiomyocyte survival	Qiao et al. (2019)
	Embryonic stem cells	FGF2	Improvement of cardiac function and promoted myocardial angiogenesis	Pang et al. (2021)
	Cardiomyocytes	miR-30d	Cardiomyocyte growth and protect against apoptosis	Melman et al. (2015)
ARDS and ALI	Endothelial progenitor cells	miR-126	Reduce inflammation, reduce myeloperoxidase activity, reduce lung injury, reduce pulmonary edema	Zhou et al. (2019)
IPF	Lung spheroid cell	miR-30a, let-7 and miR-99 family	Normalize alveolar structure, decrease collagen accumulation, decrease myofibroblast proliferation	Dinh et al. (2020)
	Macrophage	miR-142-3p	Reduce TGFβ-R1 and profibrotic genes. Present antifibrotic properties to recipient cells	Guiot et al. (2020)
	Induced pluripotent stem cell	miR-302a-3p	Supress M2 macrophages targeting, mitigate pulmonary fibrosis, reduced collagen deposition	Zhou et al. (2021)
	Human bronchial epithelial cells	miR-16, miR-26a, miR-26b, miR-141, miR-148a, and miR-200a	Reduce expression of β-catenin, attenuate cell senescence. Antifibrotic properties	Kadota et al. (2021)
COPD	hUCMSC	Not defined	Reduction of alveolar destruction, a reduction of the accumulation of immune cells in the lung, and a restoration of tissue in lung	Ridzuan et al. (2021)
	ADSC	Not defined	Attenuate airway mucus overproduction, attenuate lung inflammation, attenuate injury, inhibiting macrophage pyroptosis	Zhu et al. (2022)
CF	Human bronchial submucosal glandular cells, human adenocarcinoma alveolar basal epithelial cells	GFP-CFTR glycoprotein	Correct CFTR chloride channel function	Vituret et al. (2016)
	Stromal-derived mesenchymal stem cells	CFTR promoter (CFZF-VPR)	Activate CFTR expression	Villamizar et al. (2021)

ARDS, Acute respiratory lung injury; ALI, Acute lung injury; IPF, Idiopathic pulmonary fibrosis; COPD, Chronic obstructive pulmonary disorder; CF, Cystic fibrosis; ADSC, Adipose derived stem cells; MSC, Mesenchymal stem cells; hUCMSC, Human umbilical cord mesenchymal stem cells; HAL, Encapsulated hexyl 5-aminolevulinate hydrochloride; FGF2, Fibroblast growth factor 2; CFTR, Cystic fibrosis transmembrane conductance regulator; TGFβ-R1, Transforming growth factor β receptor 1.

## 2 EVs in atherosclerosis

### 2.1 EVs in atherosclerosis pathogenesis

Atherosclerosis is a chronic disease characterized by the accumulation of lipids and cholesterol-engorged macrophages into the arterial wall which can result in myocardial infarction or stroke after clinical complications (Lusis, 2000). High levels of EVs have been found in patients after cardiovascular events and they show differing roles during the various stages of disease: development, progression and complications of atherosclerosis (Rautou et al., 2011).The onset of atherosclerosis is mediated by the disfunction of endothelial cells (ECs) and immune cell infiltration. In a recent study of atherosclerosis, EVs isolated from monocytes were found to contain the long non-coding RNA (lncRNA) GAS5 which may be able to accelerate progression of atherosclerosis by enhancing the apoptosis of vascular endothelial cells (Chen et al., 2017). Endothelial cell-derived EVs carrying miR-19b were similarly shown to promote atherosclerosis, increasing proinflammatory contents such as TNF- α, IL-6 and decreasing anti-inflammatory IL-10 in the adipose tissue (Li C. et al., 2018). These studies demonstrate roles for EVs in promoting inflammation and implicate development of atherosclerosis, especially considering chronic inflammation is a key contributor to atherosclerosis (Bäck et al., 2019). Macrophages that are treated with ox-LDL produced EVs-that contain miR-146a. These EVs accelerate development of atherosclerosis by promoting macrophage entrapment in the vessel wall, reducing cell migration (Nguyen et al., 2018). In addition, EVs containing miR-146b, also derived from ox-LDL-treated macrophages, have been found to promote formation of neutrophil extracellular traps (NETs) by inducing oxidative stress (Zhang et al., 2019). The effects of NETosis in the initiation and progression of atherosclerosis has been discussed in an excellent review (Döring et al., 2017), and the understanding of how these EVs effect NETosis in atherosclerosis is critical to the understanding of the disease progression. Similarly, plaque accumulation accelerates atherosclerosis progression as well and it has been reported that nicotine treated macrophages release EVs containing miR-21-3p which promotes the migration and proliferation of vascular smooth muscle cells (VSMC) (Zhu et al., 2019). This illustrates one contribution of nicotine in the progression of atherosclerosis and the further risk of cigarette smoking in this disease. The accumulation of calcium phosphate salts in vessel wall, known as vascular calcification is common complication in patients with atherosclerosis (Shanahan et al., 2011). VSMC-derived EVs secretion enhances vascular calcification in response to calcium stress (Kapustin et al., 2015). In addition, EVs released from macrophages contribute to accelerated microcalcification (New et al., 2013).

### 2.2 EVs in atherosclerosis diagnosis

EVs have been examined as potential biomarkers of disease in many arenas, atherosclerosis included. By examining the cargo, specifically miRNA, contained in these EVs, it is possible to identify various disease characteristics. Lymph from atherosclerotic mice showed a higher concentration of EVs, implicating that these EVs could be biomarkers for inflammatory disease progression (Milasan et al., 2016). EVs containing specific miRNA (miR-10a-5p, miR-101-3p, and miR-24-3p, etc.), described by Jiang et al., can be used as biomarkers to predict intracranial atherosclerotic disease ischemic events (Jiang et al., 2019). In addition, release of miR-92a-3p by endothelial EVs is associated with additional atherosclerotic conditions (Liu Y. et al., 2019). Circulating EVs have been proposed as prognostic markers for major cardiovascular events (Suades et al., 2019). Circulating miR-130a-3p are associated with coronary atherosclerosis (de Gonzalo-Calvo et al., 2017). Patients with coronary atherosclerosis were observed to produce EVs that had elevated levels of miR-30e (Wang et al., 2019). These results demonstrate that EVs containing miR-130a-3p and miR-30e may have the potential as diagnostic biomarkers for coronary atherosclerosis.

### 2.3 EVs in atherosclerosis therapeutics

Inflammation is a key contributor to atherosclerosis, and as such, the alleviation of inflammation in these patients is a promising treatment for disease mitigation. Wu G. et al. (2020) developed hexyl 5-aminoevulinate hydrochloride (HAL) engineered M2 macrophage EVs with inflammation-tropism and anti-inflammatory capabilities to treat atherosclerosis. In another study, EVs produced by bone marrow-derived macrophages (BMDM-exo) containing miRNA-99a/146b/378a were able to target NF-κB and TNF-α signaling and serve as mediators anti-inflammation (Bouchareychas et al., 2020). These miRNA are increased in EVs from BMDM polarized with IL-4 (BMDM-IL-4-exo), thus these EVs may represent a therapeutic approach for atherosclerosis *via* miRNA cargo delivery (Bouchareychas et al., 2020). EVs could also be used as drug delivery systems. Late endothelial progenitor cells from atherosclerotic hamster model improved functionality by circulating EVs miRNA transfer (Alexandru et al., 2017). Subcutaneous adipose tissue stem cells (ADSC)-EVs and mesenchymal stem cells (MSC)-EVs in combination or alone with Smad2/3 siRNA were used to treat animal models of atherosclerosis which showed restoration of the elasticity of the wall of the thoracic aorta (Comariţa et al., 2022). They also showed a decreased expression of structural and inflammatory cytokines. Treatments with ADSC-EVs and MSC-EVs transfected with Smad2/3 siRNA amplified the ability to regress the inflammation mediated by the atherosclerosis process (Comariţa et al., 2022). Putting all of this together, EVs affect atherosclerosis in a variety of ways. They can increase disease severity by signaling increasing inflammation, have the ability to predict disease severity when used as biomarkers (identifying miRNA tags), and even serve as potential therapies, either by taking advantage of some of the natural anti-inflammatory capabilities of the EVs, or by engineering them to deliver specific cargos to minimize disease severity.

## 3 EVs in myocardial infarction

### 3.1 EVs in myocardial infarction pathogenesis

Myocardial infarction is a common presentation for ischemic heart disease that leads to irreversible cardiomyocyte loss (Sahoo and Losordo, 2014). The initiation of cardiovascular disease, such as myocardial infarction, can be caused by the senescence of cardiac cells, resulting in the arrest of cellular growth. There is evidence that EVs are capable of transmitting signals to nearby cells, triggering them to enter into premature senescence (Borghesan et al., 2019). In a study examining EVs from endothelial cells of patients with acute coronary syndrome, there was evidence showing these EVs led to an induction of premature endothelial senescence and thrombogenicity *via* angiotensin II (Abbas et al., 2017). This illustrates an additional role EVs play in the initiation of myocardial infarction, as these EVs from senescent endothelial cell derived deliver prosenescent message to neighbor cells, propagating early senescence in nearby cells.

### 3.2 EVs in myocardial infarction diagnosis

After myocardial infarction, dead tissue that developed from the ischemic injury is removed through cell clearance mechanisms *via* apoptotic signals. During myocardial infarction, EVs containing miR-23-27-24 result in adipocyte endoplasmic reticulum stress and endocrine dysfunction (Gan et al., 2022). Targeting these EVs may be important in the prevention of metabolic dysfunction after myocardial infarction. Conversely, measuring the expression level of cardiomyocyte-rich miR-23-27-24 can be used as disease biomarkers since they are significantly increased in the ischemic cardiomyocytes (Gan et al., 2022). Another study concluded that serum EVs containing high levels of miR-150 were associated with post myocardial infarction (Lin et al., 2019). These biomarkers are important in predicting myocardial infarctions and may improve poor prognosis.

### 3.3 EVs in myocardial infarction therapeutics

EVs have been found to modulate cardiac inflammation and possess potential uses as therapeutic tools. In a mouse model of myocardial ischemia injury, MSC derived EVs demonstrated an ability to reduce infarct size (Lai et al., 2010). Another study with MSC-derived EVs showed a reduction of infarct size by 45% compared to control treatment through an increase of ATP and NADH, decrease in oxidative stress and active pro-survival signaling which enhance cardiac function after myocardial ischemia/reperfusion injury in mice (Arslan et al., 2013). Uptake of MSC-derived EVs by neonatal mouse cardiomyocytes lead to protection against oxidative stress by substantial reduction of apoptosis, besides cardiac function was improved with promotion of angiogenesis and decrement of inflammation (Firoozi et al., 2020). Infarcted hearts injected with EVs from cardiac progenitor cells that were highly enriched with miR-210, miR-132, and miR-146a-3p showed less cardiomyocyte apoptosis, enhanced angiogenesis and improvement of cardiac function after myocardial infarction, (Barile et al., 2014). These roles represent an approach towards tissue injury repair and transplantation. EVs derived from mouse embryonic stem cells demonstrated the ability to enhance neovascularization, cardiomyocyte survival following myocardial infarction. These EVs possessed a high amount of miR-294 which promotes survival, cell cycle progression and proliferation (Khan et al., 2015). An intravenous injection of miR21-loaded CD47EVs showed improvement in cardiac morphology and functional recovery in mouse myocardial reperfusion injury models (Wei et al., 2021). The use of EVs as a drug delivery method illustrates a potential therapeutic tool for myocardial infarction. On the other hand, M2 macrophages-derived circUbe3a-containing EVs promote proliferation, migration, and phenotypic transformation of cardiac fibroblasts by targeting miR-138-5p/RhoC axis, giving an insight in the use of EVs in myocardial infarction (Wang Y. et al., 2021). Similarly, in a myocardial infarction animal model, cell apoptosis inhibition and cardiac function improvement was observed by miR-21-loaded EVs sent into cardiomyocytes and endothelial cells (Song et al., 2019). EVs have been shown to improve angiogenesis, decrease inflammation, and protect against oxidative stress, showing different approaches to treat against myocardial infarction. Macrophage-derived EV-miR-150 on myocardial infarction heart injury demonstrated cardioprotective effect by negatively regulating the TP53-IGF-1 signaling pathway (Zheng et al., 2020).

## 4 EVs in heart failure

### 4.1 EVs in heart failure pathogenesis

**FIGURE 1:**
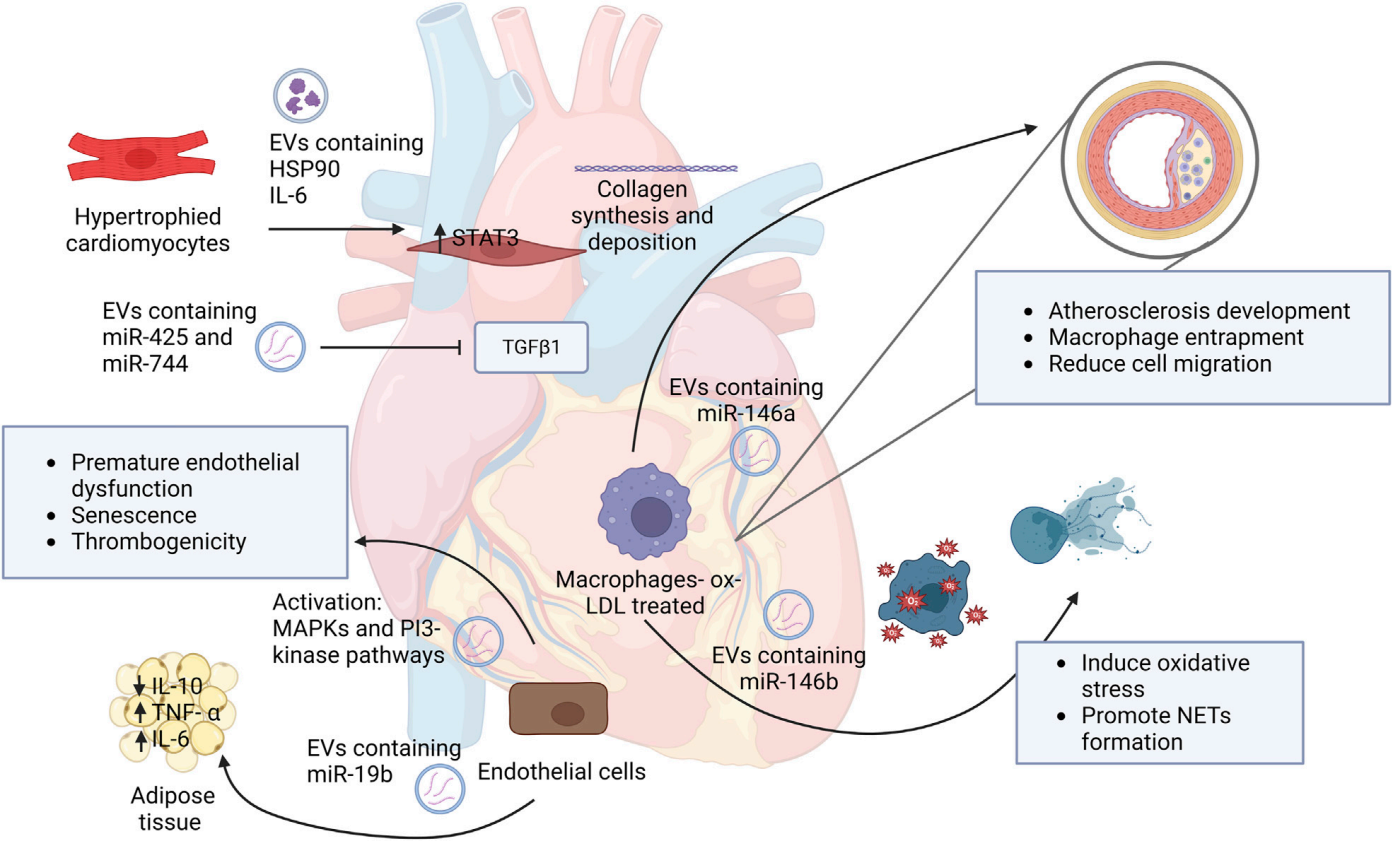
Schematic representation of EVs roles in the development and progression of cardiovascular diseases. This figure demonstrates the role of EVs and their cargo on neighbor cells and how they contribute to disease initiation. Abbreviations: HSP90, heat shock protein 90; IL-6, interleukin 6; STAT3, signal transducer and activator of transcription; MAPKs, Mitogen-activated protein kinases; IL-10, interleukin 10; TNF- α, tumor necrosis factor alpha; ox-LDL, oxidized low-density lipoprotein; NETs, neutrophil extracellular traps. Created with BioRender.com.

Heart failure (HF) is a heterogeneous syndrome that results from impairment of ventricular ejection of blood associated with symptoms such as fatigue and dyspnea (McKee et al., 1971). Currently the only treatment for HF patients who are not responding to medical therapy is transplantation. Studying the roles of EVs in HF can have a significant impact on the high morbidity and mortality of this disease. As previously stated, EVs have a major role in the pathogenesis in cardiovascular diseases, including HF. Figure 1 illustrates some of the main roles of EVs in the pathogenesis of cardiovascular diseases. A study by Wang et al. discovered EVs in plasma that showed a reduction of miR-425 and miR-744 contributed to an increase of HF severity (Wang et al., 2018). Hypertrophied myocyte-derived EVs containing heat shock protein (Hsp90) and IL-6 which are responsible for the activation of signal transducer and activator of transcription 3 (STAT-3) signaling in cardiac fibroblasts, resulting in excess collagen synthesis and deposition, (Datta et al., 2017) thus revealing a profibrotic role of EVs by their cargo.

### 4.2 EVs in heart failure diagnosis

The levels of miR-425 and miR-744 in plasma EVs contribute to heart failure severity. This association could be used as a biomarker to predict heart failure development (Wang et al., 2018). Patients who experienced HF had EVs whose miRNA ratio of miR-146a/miR-16 was higher than in patients without HF. Since miR-16 is induced in response to inflammation, the circulating EV miR-16 can be used as a biomarker for heart failure as well (Beg et al., 2017). In infarcted hearts, miR-27a, miR-28-3p and miR-34a were highly expressed in EVs, illustrating yet another group of potential biomarkers for heart infarct (Tian et al., 2018). Additionally, EVs containing miR-192, miR-194, and miR-34a were identified as regulators of HF development after acute myocardial infarction (Matsumoto et al., 2013). These EVs could be used as additional biomarkers to predict HF development after acute myocardial infarction.

### 4.3 EVs in heart failure therapeutics

There are many studies that have revealed potential therapeutic roles for EVs and HF. One such study showed miRNA-enriched EV communication between cardiac fibroblasts and cardiomyocytes which may contribute as therapeutic strategies in chronic HF by targeting Nrf2 related miRNAs (Tian et al., 2018). In another, circulating miR-30d in cardiomyocytes is released *via* vesicles in response to mechanical stress. An overexpression of miR-30d led to cardiomyocyte growth and protected against apoptosis by targeting the mitogen-associated kinase 4 in these cells (Melman et al., 2015). Further, patients with high levels of miR-30d in EVs had significantly lower mortality and these miR-30d containing EVs may be protective against 1 year mortality in HF (Xiao et al., 2017). In a mouse model of HF, it was shown that miR-30d improves cardiac function, decreases myocardial fibrosis and attenuated cardiomyocyte apoptosis (Li et al., 2021). Qiao et al. showed the importance of miR-21-5p in EVs in mediating heart repair through the enhancement of angiogenesis and cardiomyocyte survival. (Qiao et al., 2019). EVs from embryonic stem cells that contained fibroblast growth factor 2 (FGF2) were shown to attenuate transverse aortic constriction (TAC)-induced HF (Pang et al., 2021). This finding showed an improvement in cardiac function and promotion of myocardial angiogenesis by FGF2 signaling (Pang et al., 2021). It has also been observed that EVs from cardiac fibroblast-induced pluripotent stem cell (CF-iPSCs) have lower levels of miR22, indicating that those EVs are naïve of congestive heart cell memory, and could act as a potential regenerative therapy for cardiac injury (Kurtzwald-Josefson et al., 2020). Oh et al. (2020) identified upregulation of miRNAs in failing hearts. Among the upregulated miRNAs, miR-92b, miR-139 and miR-378b have been identified to be anti-hypertrophic, and miR-139, miR-378a and miR-345 are known to be anti-fibrotic. Therefore, these anti-hypertrophic and anti-fibrotic miRNAs in EVs may play an important role in cardioprotection. Zhong et al. (2021) showed that human umbilical cord mesenchymal stem cell-derived EVs (hUCMSC-EVs) are capable of inhibiting doxorubicin (DOX)- induced HF by inhibiting cardiomyocyte oxidative stress, apoptosis and the expression of the NOX4 protein. EVs containing miR-100-5-p play a role similar to hUCMSC-EVs in reducing the oxidative stress and apoptosis. This suggests that hUCMSC-EVs are capable of inhibiting DOX-induced HF potentially *via* the miR-100-5p/NOX4 pathway (Zhong et al., 2021). Additionally, the presence of increased miR-132 expression in EVs was shown to enhance the anti-oxidative stress and anti-apoptotic effects (Liu. X et al., 2018), thus making them a potential alternative treatment against the development of HF.

## 5 EVs in acute respiratory distress syndrome (ARDS) and acute respiratory lung injury (ALI)

### 5.1 EVs in ARDS and ALI pathogenesis

**FIGURE 2:**
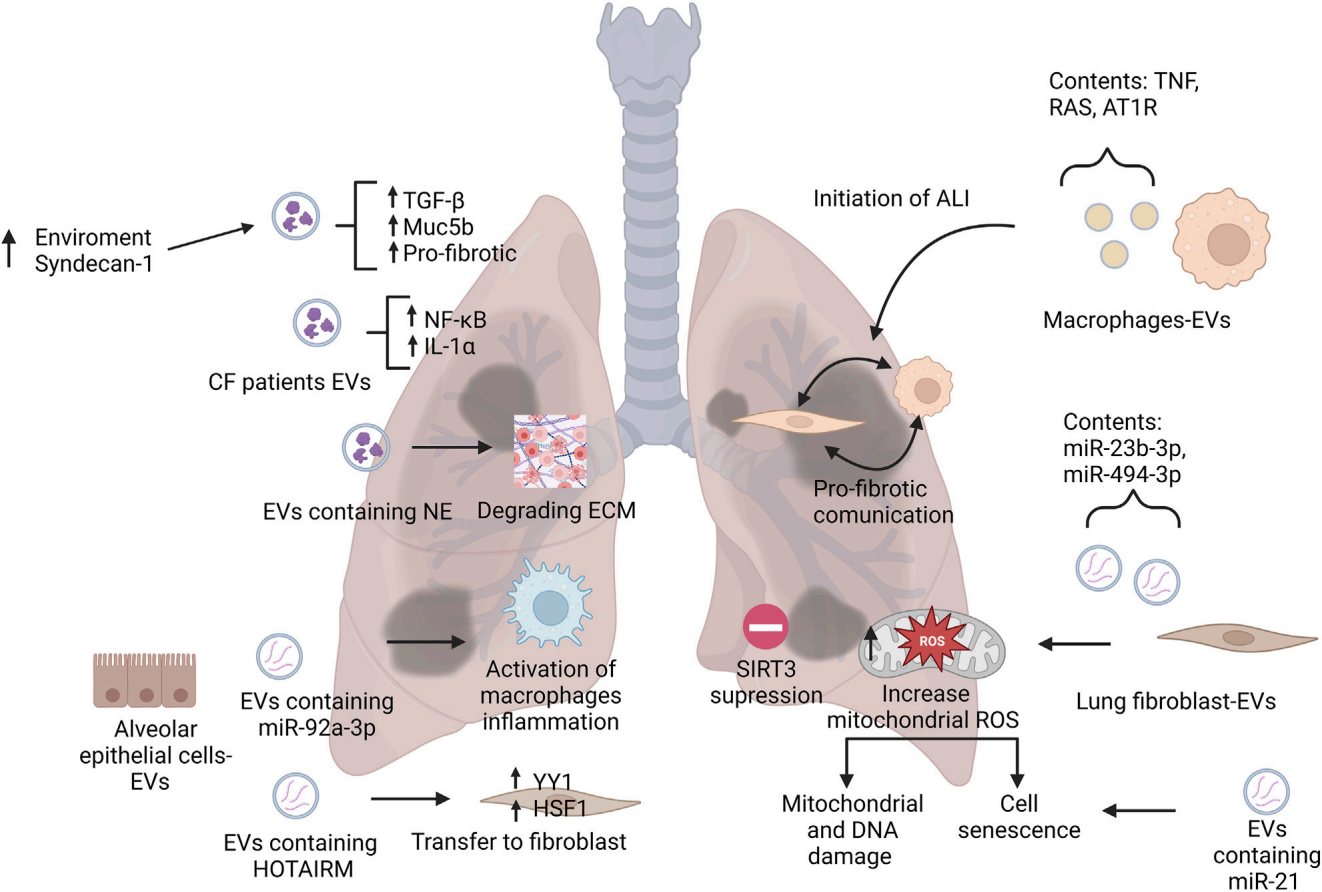
Representation of EVs roles in pathogenesis of pulmonary diseases. This figure illustrates the main roles of EVs on the initiation and contribution to pulmonary disease by modulating the environment and nearby cells. Abbreviations: CF, cystic fibrosis; NE, neutrophil elastase; TGF- β, tumor necrosis factor beta; IL-α, interleukin alpha; NF-κB, nuclear factor kappa B; ECM, extracellular matrix; HSF1, heat shock factor 1; ALI, acute lung injury; SIRT3, sirtuin 3; TNF, tumor necrosis factor; RAS, renin-angiotensin system; AT1R, angiotensin II type 1 receptor; ROS, reactive oxygen species. Created with BioRender.com.

Acute respiratory distress syndrome (ARDS) and acute lung injury (ALI) are characterized by inflammation that cause diffuse alveolar damage (Dushianthan et al., 2011). The role of extracellular vesicles in ALI remains mostly unknown, however, there have been a few studies demonstrating their effects. Figure 2 illustrates EVs contribution to disease progression and initiation of pulmonary diseases such as ARDS and ALI. One such study of macrophage derived EVs suggests that they harbor tumor necrosis factor (TNF), which may play a role in the initiation of ALI (Soni et al., 2016). Monocyte-derived EVs containing gasdermin D are enriched in patients with ALI and they showed a critical role in caspase 1-mediated endothelial injury (Mitra et al., 2018). Alveolar epithelial cell (AEC)-derived EVs were shown to activate alveolar macrophages, inducing inflammation which was mediated by miR-92a-3p in ALI (Liu. X et al., 2021). These findings indicate the potential pathogenetic capability of these monocyte and AEC-derived EVs in ALI.

### 5.2 EVs in ARDS and ALI diagnosis

Extracellular vesicles derived from alveolar macrophages that contain TNF make good candidates for biomarkers of ALI (Soni et al., 2016), as do alveolar epithelial cell-derived EVs that are high in miR-92a-3p. (Liu. F et al., 2021). High concentrations of endothelial EVs that contain nitrated sphingosine-1-phosphate receptor-3 have been found to be associated with high mortality in patients with sepsis and ALI (Sun et al., 2012). These EVs also are released during inflammatory lung states which can represent a biomarker for ALI outcomes (Sun et al., 2012). Identifying these cargos in EVs could help to predict the stage of disease, such as sepsis or inflammation.

### 5.3 EVs in ARDS and ALI therapeutics

The inflammatory disorder is a key for the development of ALI. Inflammatory response helps with the recruitment of the immune cells and release of cytokines. This can damage the pulmonary epithelial cells and targeting this inflammation response may represent an important approach to minimize disease pathology. As previously mentioned, EVs have been shown to combat this inflammatory response associated with multiple diseases. EVs from endothelial progenitor cells protect against acute lung injury through the delivery of miR-126, thus reducing inflammation, myeloperoxidase activity, lung injury score and pulmonary edema (Zhou et al., 2019). MSC- derived EVs injected into the tail veins of mice used in acute lung injury model reduced levels of neutrophils and macrophages in the bronchoalveolar lavage fluid (BALF) as well as lowering the levels of inflammatory protein-2 (MIP-2) (Li et al., 2015). In another study of phosgene-induced ALI rat model, Xu et al. (2019) found that intratracheal administration of MSC-derived EVs reduced the level of TNF-α, IL-1β, and IL-6, all of which modulated the inflammation in ALI. All of these results show a protective effect of MSC-derived EVs on acute lung injury. Long coding RNAs (lncRNA), such as lncRNA-p21, induced downregulation of miR-181 and could suppress cell apoptosis and alleviate lung tissue injury on sepsis induced- ALI (Sui et al., 2021). MSC-derived EVs mitigate ALI by transferring miR-27a-3p to alveolar macrophages which act to target NFKB1 and regulate M2 macrophage polarization (Wang et al., 2020). Bone marrow MSC-derived EVs improved the survival rate of ALI rats by alleviating lung pathological changes and attenuating inflammatory response with the enrichment of miR-384-5p found in the EVs (Liu. X et al., 2021). MSC-derived EVs demonstrated the ability to reduce lung injury and restore mitochondrial respiration in the lung tissue in the ARDS environment (Dutra Silva et al., 2021). All these findings represent potential therapeutic approaches for EVs in the treatment of ALI/ARDS.

## 6 EVs in idiopathic pulmonary fibrosis (IPF)

### 6.1 EVs in IPF pathogenesis

Idiopathic pulmonary fibrosis (IPF) is characterized by progressive dyspnea and high decline of lung function (Rigante and Stabile, 2004). The role of EVs on the pathogenesis of IPF has not yet been clear. Parimon et al. (2019) showed that EVs modulate profibrotic pathways which lead to pulmonary fibrosis due to the overexpression of syndecan-1 in the profibrotic environment. These results suggest that cargo and roles of EVs are regulated by their environment. Macrophages are involved in the pathogenesis of IPF by activation of lung fibroblasts (Li Y. et al., 2018). A study where macrophage derived EVs containing Angiotensin II type 1 receptor (AT1R) were injected to bleomycin (BLM)—induced lung fibrosis mice showed enhancement of macrophage infiltration, increasing pro-fibrotic communication between the macrophages and fibroblasts (Sun et al., 2021). Understanding these mechanisms that effect pathogenesis can provide therapeutic approaches to reverse pathological changes. Pulmonary fibrosis is considered a result from aberrant wound healing which leads to fibroblast accumulation and deposition of excessive amounts of extracellular matrix components, specially, collagen (Deng et al., 2020). AEC-derived EVs can accelerate IPF progression by delivering of HOTAIRM1 which facilitates the proliferation and trans-differentiation of lung fibroblasts as well as extracellular matrix remodeling (Chen et al., 2022). Additionally, lung fibroblast-derived EVs from IPF patients with high levels of miR-23b-3p and miR-494-3p increased the production of mitochondrial reactive oxygen species inducing lung epithelial cell senescence (Kadota et al., 2020). Interestingly, this contribution that EVs have on the progression and development of IPF could also represent a strategy against IPF.

### 6.2 EVs in IPF diagnosis

In a BLM-induced lung fibrosis mouse model, serum EVs with miR-21-5p were significantly higher compared to the control, its baseline was correlated with the rate of decline in vital capacity and associated with mortality (Makiguchi et al., 2016). The high level of EV miR-21-5p showed a poorer prognosis suggesting that it can be used to predict progression and mortality (Makiguchi et al., 2016). These findings put EV miR-21-5p as a potential prognostic biomarker for IPF. In patients with IPF, miR-125b, miR-128, miR-21, miR-100, miR-140-3p and miR-374b where all upregulated, indicating they, too, can be used as potential biomarkers (Liu. B et al., 2018). Njock et al. found EV associated miR-142-3p in sputum-derived EVs of patients with IPF revealing a potential biomarker for diagnosis and disease severity (Njock et al., 2019).

### 6.3 EVs in IPF therapeutics

Many studies have demonstrated the role of EVs with anti-fibrotic properties towards IPF. Human bronchial epithelial cell-derived EVs possess anti-fibrotic properties *via* miRNA-mediated inhibition of TGF-β-WNT crosstalk (Kadota et al., 2021). On the other hand, menstrual blood-derived stem cell (MenSCs) EVs showed immunosuppression and antifibrosis (Sun et al., 2019). This study showed EV associated Let-7 from MenSC minimizes pulmonary fibrosis by regulating reactive oxygen species, mtDNA damage and NLRP3 inflammasome activation (Sun et al., 2019). MiR-16 *via* the mTORC2 pathway showed antifibrotic properties as well (Inomata et al., 2021). EVs from hUCMSC demonstrated the ability to alleviate pulmonary fibrosis in mice by significantly reducing pulmonary index, collagen deposition and lung tissue pathologies (Yang et al., 2020). Mice treated with hUCMSC-derived EVs can inhibit the epithelial mesenchymal transition activated by TGF-β1/Smad2/3 signaling pathway (Yang et al., 2020). After treatment in mice with iPSC-derived EVs the level of miR-302a-3p was increased (Zhou et al., 2021). MiR-302a-3p can suppress M2 macrophages targeting and consequently mitigate pulmonary fibrosis (Zhou et al., 2021). EVs isolated from macrophages reduced the expression of transforming growth factor β receptor 1 (TGFβ-R1) and profibrotic genes by the overexpression of miR-142-3p in alveolar epithelial cells (Guiot et al., 2020). The antifibrotic properties of miR-142-3p suggest that EVs from macrophages may act against IPF progression. Lung tissue loses the ability to facilitate gas exchange due to the thickening of fibrosis, therefore, the study of treatments to approach this can improve the prognosis of IPF patients. Inhalation of lung spheroid cell-EVs can attenuate BLM-induced fibrosis by normalizing alveolar structure and decreasing collagen accumulation (Dinh et al., 2020), thus showing that targeting pulmonary fibrosis progression and deposition of collagen is a novel therapeutic approach for IPF.

## 7 EVs in chronic obstructive pulmonary disorder (COPD)

### 7.1 EVs in COPD pathogenesis

Chronic obstructive pulmonary disorder (COPD) is a chronic respiratory disorder characterized by airway obstruction and emphysema. Cigarette smoking is one of the most common risk factors (Barnes et al., 2015). Being that COPD is a disease that has such high global morbidity and mortality, it has attracted a lot of research on the roles that EVs play in pathogenesis as well as therapeutics. Previously, our group described a novel pathogenic entity, the neutrophil derived EV with catalytically active neutrophil elastase (NE) on the surface capable of degrading extracellular matrix (ECM) (Genschmer et al., 2019). These EVs were also found in the BALF of subjects with COPD and the purified EVs could transfer a COPD-like emphysema phenotype when administered intratracheally to naïve mice. As previously mentioned, since cigarette smoke is a common risk factor of COPD, the role of EVs in this model is an area of interest. Cordazzo et al. (2014) showed that when mononuclear cells were exposed to cigarette smoke extract (CSE), this induced a rapid increase in [Ca (2+)]i mobilization and the release EVs. When these CSE induced EVs were incubated with lung epithelial cells, an upregulation of pro-inflammatory mediator synthesis from these epithelial cells were observed, illustrating a pro-inflammatory communication between mononuclear cells and lung epithelial cells *via* CSE induced EV release. In a similar study of the effects of CSE on bronchial epithelial cell EV release, Fujita et al. (2015), EV mediated cellular communication between human bronchial epithelial cells and lung fibroblasts was observed, demonstrated *via* an upregulation of EV miR-210 expression promoting lung fibroblast differentiation and upregulating the autophagy machinery. CSE-treated alveolar epithelial cell release EVs promoting the polarization of M1 macrophages and aggravating impairment in pulmonary function and lung injury in mice trough TREM-1 expression (Wang L. et al., 2021). These increases in M1 macrophage polarization by CSE EVs is of interest because an increase in M1 macrophages and a decrease of M2 macrophages in the small airway of COPD patients has been observed (Eapen et al., 2017). These previous studies examine the potential contribution of M1 macrophages polarization to the progression of COPD and suggest that exploring the effect of macrophage polarization could contribute to potential targets for treatment or therapeutics for COPD patients. Conversely, a study by He et al. (2019) discovered that a compensatory role of EVs produced by cells exposed to CSE exist. Serum EVs of COPD patients were found to have elevated amounts of miR-21. However, they noted that when bronchial epithelial cells (BEAS-2B) were treated with CSE, they produced EVs with lower miR-21, and that these EVs alleviated M2 macrophage polarization. Not only do these studies indicate that miR-21 in serum EVs could be used as a biomarker for COPD, but that the use of EVs low in miR-21 from bronchial epithelial cells (BECs) could be a potential therapeutic. On the other hand, cell senescence is a key for the development of COPD. As previously stated, there is an upregulation of EV miR-21 in COPD patients, and it has been shown that miR-21 is high in cell senescence and contributes to the induction of senescence cell-cycle arrest (Dellago et al., 2013).

### 7.2 EVs in COPD diagnosis

Additional miRNAs have been observed in COPD patients that have the potential to be used as disease biomarkers. Sundar et al. (2019) noted an upregulation of miRNAs such as miR-22-3p, miR-99a-5p, miR-151a-5p, miR-320b and miR-320d in EVs isolated from COPD smokers’ plasma. Additionally, observations of the expression of miR320b and miR-22-3p in the EVs from BALF-derived from COPD patients as compared to the non-smokers has been implicated as potential identifiers of disease (Kaur et al., 2021). While miR-22-3p has been reported to suppress histone deacetylase 4 (HDAC4) and promote an increase in Th17 causing an increase in IL-6 and TNF which resulting in disruption of alveolar walls and causing emphysema in mice that were exposed to cigarette smoke (Lu et al., 2015), the levels of miR-22-3p varies among patients depending on their history of smoking or exposure of biomass smoke (Velasco-Torres et al., 2019). These data not only show the role of miR-22-3p in the regulation of emphysema but may act as a biomarker to reveal the progression of the disease in patients.

### 7.3 EVs in COPD therapeutics

There is still a lack of treatment that can effectively reverse the progression of COPD. However, EVs have been studied along with MSC-based therapies that hold promise for the treatment of COPD by improving lung function and survival (Cruz and Rocco, 2020). EVs secreted by injured alveolar epithelial type II cells could help promote the proliferation and migration of MSC, along with upregulating expression levels of genes related to mitochondrial synthesis (Song et al., 2021). These EV-mediated communication pathways could provide new targets for MSC-based therapies. Furthermore, a combination of MSC and EVs have a protective effect against mitochondrial dysfunction caused by exposure to cigarette smoke (Maremanda et al., 2019). Treatment with hUCMSC-EVs caused a reduction of alveolar destruction, a reduction of the accumulation of immune cells in the lung, and a restoration of tissue on rat lung after cigarette smoke exposure (Ridzuan et al., 2021). Mitochondrial dysfunction has a known role in the pathogenesis of COPD (Nam et al., 2017), and therefore, targeting this dysfunction could be a potential target for treatment of this disease. Similarly, treatment with human adipose-derived stem cells-derived EVs (ADSCs-Exo) attenuated the cigarette smoke induced lung overproduction of mucus, lung injury and, inflammation by inhibiting alveolar macrophage pyroptosis (Zhu et al., 2022). hUCMSC-EVs and ADSCs-Exo also showed anti-inflammatory effects which could potentially be used as a treatment for COPD.

## 8 EVs in cystic fibrosis (CF)

### 8.1 EVs in CF pathogenesis

Cystic fibrosis (CF) is an autosomal recessive respiratory genetic disease caused by mutation in the cystic fibrosis transmembrane conductance regulator (CFTR) gene (Riordan et al., 1989). The CF disease phenotype is characterized by chronic bacterial infection, airway obstruction, inflammation, and short life expectancy (Davis et al., 1996). CF sputum-derived EVs have displayed a high pro-inflammatory effect *in vivo* in the mouse lung (Porro et al., 2013). Rollet-Cohen and colleagues identified high levels of proteins in EVs from CF patients that were involved in the activation of NF-κB pathway suggesting that EVs are involved in the proinflammatory propagation of CF (Rollet-Cohen et al., 2018). Some of the proteins contained in these EVs were antioxidant proteins (Superoxide-dismutase, Glutathione peroxidase-3, Peroxiredoxin-5) and proteins involved in leukocyte chemotaxis (Rollet-Cohen et al., 2018). It has also been noted that high levels of IL-1 proinflammatory mediators increased in the CF airway fluid (Tang et al., 2012; Forrest et al., 2022). Previous studies have reported a role of IL-1β in CF inflammatory disease since it can generate fever and recruit inflammatory effector cells (Weber et al., 2010). Similarly, EVs from activated neutrophils can deliver active caspase-1 to tracheal epithelial cells and induce the release of IL-1α (Forrest et al., 2022). EVs can cause CF inflammatory pathogenesis by modulating the recruitment of proinflammatory mediators.

### 8.2 EVs in CF diagnosis

EVs from CF patients have primarily been isolated from patient sputum samples and is considered a non-invasive method for detecting lung infection. High levels of EVs containing CD66b+ (a neutrophil marker) have been found in the sputum of CF patients (Porro et al., 2010). In a study by Useckaite et al. (2020) it was found that EVs have differential protein expression in different ages of patients, which can be used as biomarkers. Obtaining EVs from sputum as a biomarker leads to potential non-invasive diagnosis for this disease and can provide information about the progression of the disease.

### 8.3 EVs in CF therapeutics

Additionally, therapies targeting the function of CFTR have been studied with the goal of improving the prognosis of CF patients. Various studies have used EVs as vehicles to target CFTR function. Zinc finger protein activators have been studied as therapeutic tool to activate gene expression (Scott et al., 2021). Stromal-derived MSC were engineered to produce EVs that contain CFTR Zinc Finger Protein fusion with transcriptional activation domains to target CFTR promoter and activate transcription (Villamizar et al., 2021). Treatment with these resulted in activation of CFTR transcription in Human Bronchial Epithelial cells (HuBEC). They also found that they can pack CFZF-VPR into the MSC EVs and deliver to HuBEC to activate CFTR expression. EVs used as vehicles to deliver exogenous CFTR glycoprotein and mRNA (GFP-CFTR) showed the capacity to package and deliver both of them to cells in order to correct the CFTR chloride channel function (Vituret et al., 2016). This suggests that these EVs can act as functional correctors of a genetic defect. As mentioned before, the expression of proinflammatory cytokines has a role in the pathogenesis of CF. Targeting the expression of these cytokines may have a potential therapeutic role. A study with human lung MSCs-derived EVs showed that they can exhibit anti-inflammatory potential in *in vitro* CF model reducing the proinflammatory cytokine expression and activating PPARγ, a transcription factor controlling anti-inflammatory and antioxidant mechanisms (Zulueta et al., 2018). While CF is a genetic disease resulting in aberrant function of a chloride channel, EVs show positive potential as possible therapeutics to alleviate inflammation and maybe even deliver functional CFTR to diseased cells.

## 9 EVs in viral infection related to cardiopulmonary diseases

There are myriad viral infections that are related to, can cause, and can complicate cardiopulmonary disease. We will discuss recent EV research relating to two of these viruses, Influenza and acute respiratory syndrome (COVID-19), caused by SAR-CoV-2. Both infections cause severity of virus-induced lung damage (Flerlage et al., 2021). It has been shown that EVs have a role in antigen presentation (Raposo et al., 1996) and during viral infection, extracellular vesicles become presenting vectors of viral material (Pesce et al., 2021). Therefore, exploring the role of EVs in influenza virus and COVID-19 infection has been attracting significant attention. EVs have shown immunomodulatory effects from highly pathogenic avian influenza virus (HPAIV) HPAIV H5N1- in an infected chicken model. Macrophage, fibroblast, T cell and B cell lines were treated with serum isolated EVs from H5N1-infected chickens, resulting in the expression of proinflammatory cytokines, such as IFN-γ, IL-1β, and CXCL8 (Hong et al., 2022). Additionally, in a mouse model of influenzae, BALF-derived EVs containing miR-483-3p were highly increased in influenza virus-infected mice (Maemura et al., 2020). miR-483-3p-enriched EVs derived from type II pneumocytes potentiated the expression of proinflammatory cytokine genes. Additionally, miR483-3p potentiated the expression of type I interferon, as well as targeting RNF5, a regulator of the RIG-I signaling pathway (Maemura et al., 2018). This suggesting that miR-483-3p is involved in the pathogenesis of H5N1 virus infection, mediate the antiviral response and can be used as a potential biomarker. The most targeted genes were related to MAPK signaling pathway which gives a better understanding of immune response and could help to find biomarkers for resistance. EVs extracted from the supernatant of Influenza A virus (IAV)-infected cells were shown to promote the recruitment and polarization of more peritoneal macrophages than the normal group *via* autophagy-EV dependent pathway (Xia et al., 2022). This provides a potential mechanism for how IAVs influence pathogenesis by the recruitment of M1 macrophage polarization, and through further research into the disruption of this cascade, can potentially be used as a therapeutic in Influenza A treatment.

EVs released into the airway during influenza virus infection can change their composition during infection. They can trigger pulmonary inflammation and carry viral antigen that can drive the induction of a cellular immune response (Bedford et al., 2020). Attachment factors for influenza like, α2,3 and α2,6-linked sialic acids, were present on the EV surface which can neutralize the virus. Similarly, it was identified α2,3- and α2,6-linked sialic acid positive EVs in BALF which have shown anti-influenza activity (Suptawiwat et al., 2017). These EVs showed an antiviral innate immune defense against influenza virus infection by preventing the binding and entrance to host cells and by trapping viral particles. Influenza virus infection cause cell apoptosis, leading to biogenesis of has-miR-1975 which is delivered into EVs and engulfed by neighbor cells (Liu Y. M. et al., 2019). Has-miR-1975 induces interferon production which inhibits the virus replication, revealing an influenza antiviral mechanism through exosome delivery.

Lung inflammation is a hallmark of COVID-19. Mice that were administered EVs from SARS-CoV-2 exposed lung epithelial cells developed their own inflamed lung tissue. These EVs were taken up by lung macrophages leading to the activation of NF-κB and inflammatory cytokines (Teng et al., 2021). These EVs displayed their role in the development of lung inflammation after COVID-19 infection. Another study demonstrated that circulating EVs are modulated during COVID-19 infection and might be involved in pathogenesis by modulation of immune response, inflammation and activation of coagulation and complement pathways (Barberis et al., 2021). This suggests a significant role for EVs in the mechanisms associated with tissue damage and multiple organ dysfunctions. Viral material in the EV cargo showed the potential use for cell-cell communication to spread infection in the host and the presence of potential biomarkers for the severity of the disease. mRNA vaccines directed at the SARS-CoV-2 spike protein resulted in development of Abs and protective immunity (Bansal et al., 2021). They showed an induction of humoral and cellular immune responses after immunization with EVs carrying spike protein. A clinical trial using EVs derived from allogenic bone marrow mesenchymal stem cells restored oxygenation, downregulated cytokine storm and reconstitute immunity when administered to COVID-19 patients (Sengupta et al., 2020).

## 10 Discussion

**FIGURE 3:**
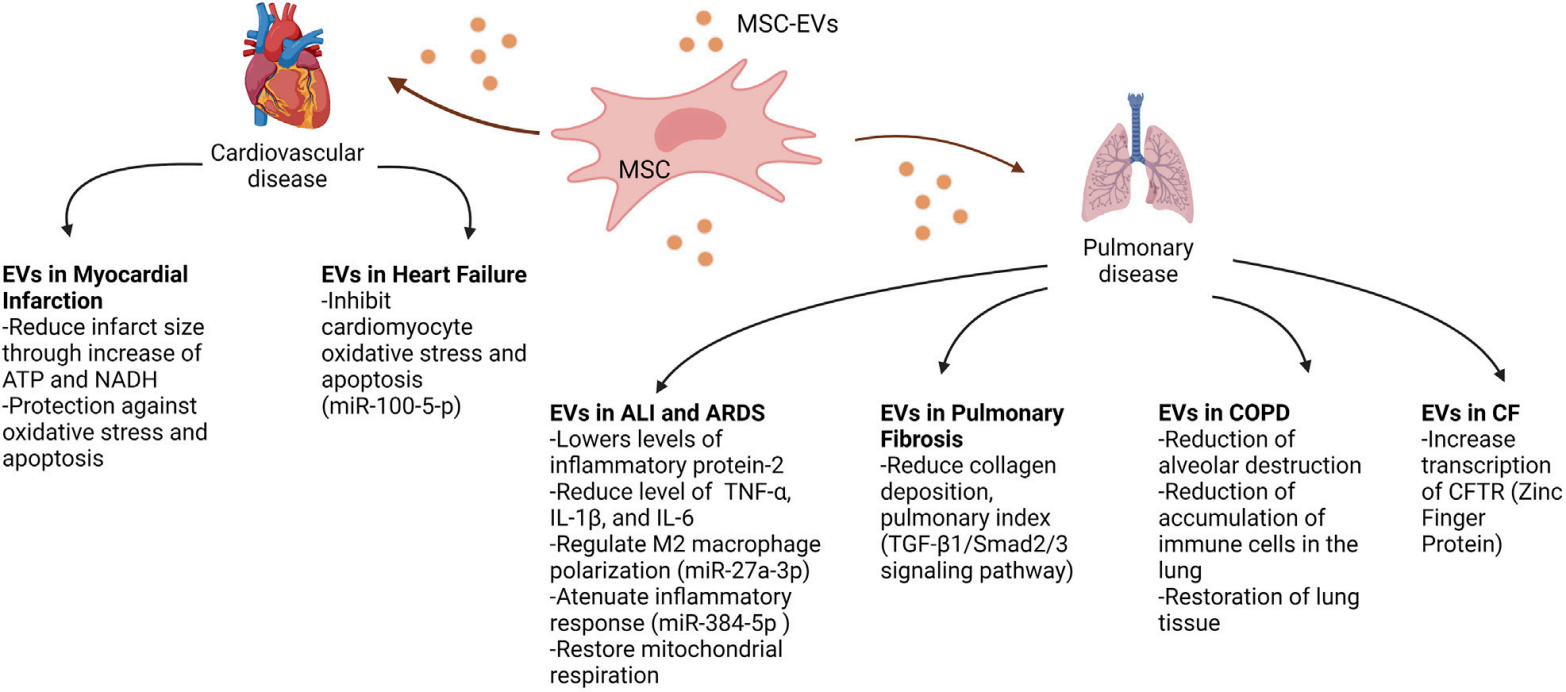
Therapeutic potential of mesenchymal stem cell-derived EVs on cardiopulmonary diseases. This figure demonstrates the various potential therapeutic effects of MSC-EVs on recipient cells, improving the phenotypic outcomes of diseases such as myocardial infarction, heart failure, acute lung injury (ALI), acute respiratory distress syndrome (ARDS), pulmonary fibrosis, chronic obstructive pulmonary disease (COPD) and cystic fibrosis (CF). Abbreviations: MSC, mesenchymal stem cell; EVs, extracellular vesicles; ATP, adenosine triphosphate; NADH, nicotinamide adenine dinucleotide; TNFα, tumour necrosis factor alpha; IL1β, Interlukin 1 beta; IL-6, Interlukin 6; TGF-β1, transforming growth factor beta 1; CFTR, cystic fibrosis transmembrane conductance regulator. Created with BioRender.com.

Extracellular vesicles hold a wealth of insight into disease pathogenesis, as biomarkers for disease states, and as potential to deliver therapeutics to halt/reverse disease progression. EVs have been used as biomarkers for various cancers for some time now (Wong and Chen, 2019), and because of this, EVs are quickly growing to become useful biomarkers to help diagnose various other conditions, including some cardiopulmonary diseases, as specified earlier. However, the use of EVs as biomarkers is just the tip of the iceberg for their usefulness in understanding cardiopulmonary disease pathogenesis and therapeutic discovery. In COPD, for example, EVs with proteolytic enzymes on their surface have been shown to be pathogenic entities on their own (Genschmer et al., 2019). But it is the potential for use as therapeutics that is the most exciting aspect of EV research in cardiopulmonary diseases. It has been shown that in the study of aging, and age related conditions, EVs from the blood of younger donors can have an effect on restoration of aged tissue (Sahu et al., 2021). These, coupled with other studies previously mentioned, show that EV cargo is dependent upon the disease state and can be modified by the microenvironment. In the study of EVs as potential therapeutics in cardiopulmonary disease, EVs mesenchymal stem cells offer a wealth of promise in potential therapy. As seen in Figure 3, we review the potential that EVs from MSC offer in relation to various cardiopulmonary diseases. This represents the effects of EVs in tissue or a certain disease may be due to the origin of the cells that they are derived from. In addition, EVs provide a vehicle for additional therapies, like full length CFTR, or other proteins/molecules that may be malformed due to genetic mutations. Comprehending these mechanisms and pathways in which EVs can cause, or potentially cure, disease will contribute to these therapeutic approaches. The expansion in therapeutic avenues imply a better prognosis for these diseases that are the biggest cause in mortality and morbidity worldwide.
